# Overexpression of miR-126 sensitizes osteosarcoma cells to apoptosis induced by epigallocatechin-3-gallate

**DOI:** 10.1186/1477-7819-12-383

**Published:** 2014-12-16

**Authors:** Liangdong Jiang, Cheng Tao, Aiyong He, Xiaojie He

**Affiliations:** Department of Orthopedics, The Second Xiangya Hospital, Central South University, No. 139 Middle Renmin Road, Changsha, Hunan 410011 P.R. China; Children’s Medical Center, The Second Xiangya Hospital, Central South University, No. 139 Middle Renmin Road, Changsha, Hunan 410011 P.R. China

**Keywords:** Osteosarcoma, miR-126, Epigallocatechin-3-gallate, Sensitization, Apoptosis

## Abstract

**Background:**

miR-126 plays an important role in the proliferation, invasion, migration, and chemotherapeutics resistance in cancer. Epigallocatechin-3-gallate (EGCG), as the major polyphenolic constituent present in green tea, is a promising anticancer agent. However, the role of miR-126 in EGCG anticancer remains unclear. Here, we investigated the effects of miR-126 and EGCG on cell viability, apoptosis, cell cycle distribution of osteosarcoma cells and the sensitization of miR-126 on osteosarcoma cells to EGCG.

**Methods:**

The cell viability, apoptosis and cycle distribution were analyzed using MTT assay and flow cytometry.

**Results:**

Our results showed that EGCG (0.025, 0.05, 0.1, 0.2 g/L) suppresses proliferation of osteosarcoma MG63 and U2OS cells in a concentration-dependent and time-dependent manner and the inhibitory effects of 0.05 g/L EGCG on U2OS cells were roughly equivalent to 20 μM cisplatin (DDP); miR-126 could promote apoptosis and inhibit proliferation in U2OS cells but without significant effects on cell cycle G1 phase arrest; EGCG suppressed proliferation of U2OS cells through induction of cell cycle G1 arrest and apoptotic death; overexpression of miR-126 enhanced the inhibitory effects of EGCG on proliferation in U2OS cells via promotion of apoptosis.

**Conclusions:**

Our results demonstrate that enhanced expression of miR-126 increased the sensitivity of osteosarcoma cells to EGCG through induction of apoptosis.

## Background

Osteosarcoma is a primary malignant bone tumor with high morbidity that occurs mainly in children and adolescents. Multiple options for the treatment of osteosarcoma have been described, including chemotherapy, radiation, and so on, however, therapeutic efficacy is typically transient and mostly absent with advanced disease [1]. Therefore, the need for more rational approaches to osteosarcoma therapy is essential.

Epigallocatechin-3-gallate (EGCG) is the most abundant catechin in green tea, showing anti-inflammatory, antioxidant, anticancer and immunomodulatory activities [2–5]. It has been shown that EGCG has extensive anticancer activities, including in breast cancer [6], cervical cancer [7], lung cancer [8], prostate cancer [9], colon cancer [10], head and neck cancer [11], gastric cancer [12], ovarian cancer [13], even cancer stem cells [14], and so on. It was reported that combined administration of EGCG and interleukin 1 (IL-1) receptor antagonist could efficiently decrease IL-1-induced tumorigenic factors, leading to reduction in angiogenesis and invasiveness in human osteosarcoma cells [15]. It suggests that a combination of EGCG and interfering approaches against factors that play a crucial role in tumor progression is a promising strategy for improving therapeutic efficacy, including osteosarcoma.

MicroRNAs (miRs), a class of 22-nucleotide noncoding RNAs, have emerged as critical components of gene-regulatory networks controlling numerous important pathophysiological processes, including the initiation and progression of tumors. Studies on miRs have opened new avenues for both the diagnosis and treatment of cancer. Many miRs, [16–18] including miR-126 [19], are found to be involved in the proliferation, invasion, migration, and drug resistance [20] in osteosarcoma cells. It was found that miR-126 was consistently underexpressed in osteosarcoma tissues and cell lines and functioned as a tumor suppressor in osteosarcoma [19]. Studies showed that miR-126 could enhance the sensitivity of lung cancer [21] and cervical cancer [22] cells to anticancer agents. However, whether miR-126 can sensitize osteosarcoma cells to EGCG remains to be elucidated.

In this study, the effects of miR-126 and EGCG on the proliferation, apoptosis and cell cycle in osteosarcoma cells were investigated. Our results showed that miR-126 could enhance the sensitivity of osteosarcoma U2OS cells to EGCG, providing novel approaches or targets for reducing drug resistance in cancer.

## Methods

### Cell culture and stimulation

MG63 and U2OS cell lines (American Type Culture Collection (ATCC), USA) were cultured in Dulbecco’s modified Eagle’s medium (DMEM) culture medium at 5% CO_2_. Cells were treated with EGCG (0.025, 0.05, 0.1, 0.2 g/L) for 24, 48 and 72 hours, or EGCG (0.05 g/L), cisplatin (DDP, 20 μM) or rapamycin (RAPA, 100 nm) for 48 hours as indicated. To investigate the roles of miR-126 in U2OS cells, the lentiviral vectors comprising pre-miR-126 or anti-miR-126 were constructed and used to infect U2OS cells, establishing stable U2OS cell lines overexpressing or silencing miR-126.

### Cell proliferation assay

Cells treated with indicated reagents or samples in exponential growth were plated at a final concentration of 2 × 10^3^ cells per well in 96-well plates. The viability of cells was evaluated by 3-(4,5-dimethylthiazol-2-yl)-2,5-diphenyltetrazolium bromide (MTT) assay after 24, 48 and 72 hours of seeding. The optical density at 570 nm (OD570) of each well was measured with an enzyme-linked immunosorbent assay (ELISA) reader (ELX-800 type, BioTek Winooski, VT, USA).

### Cell apoptosis assay

After being incubated in the presence of EGCG or RAPA for 48 hours, cells were harvested and treated with fluorescein isothiocyanate (FITC, BioVision, Mountain View, CA, USA) according to the manufacturer’s instructions. Cell apoptosis was detected by analyzing Annexin V-FITC binding by flow cytometry (Ex = 488 nm; Em = 530 nm) using a FITC signal detector and a propidium iodide (PI) signal detector.

### Cell cycle analysis

The cells were digested with trypsin (Auragene Bioscience Corporation, Changsha, China) and collected after treatment for 48 hours, and washed with phosphate-buffered saline (PBS) twice. The cells were resuspended in PBS and then fixed in 70% ethanol at 4°C for 18 hours. The cells were washed with PBS and resuspended in Staining Solution (50 μg/mL of PI, 1 mg/mL of RNase A, 0.1% Triton X-100 in PBS). The stained cells (1 × 10^5^) were then analyzed with a flow cytometer (Beckman Coulter, Brea, CA, USA).

### Statistical analysis

Data were expressed as mean ± standard deviation (SD) from at least three separate experiments. Statistical analysis was carried out using SPSS 15.0 software (SPSS Inc, Chicago, IL, USA). The difference between two groups was analyzed by the Student’s *t* test. A value of *P* <0.05 was considered statistically significant.

## Results

### EGCG inhibits proliferation of osteosarcoma cells

To investigate the effects of EGCG on proliferation of osteosarcoma cells, the human osteosarcoma U2OS and MG63 cells were treated with different concentrations (0.025, 0.05, 0.1, 0.2 g/L) of EGCG for 24, 48, or 72 hours, respectively. The MTT results showed that the relative inhibitory rate of EGCG on U2OS and MG63 cells increased with enhancement of its treatment concentration and time. It suggests that EGCG suppresses proliferation of osteosarcoma cells in a concentration-dependent and time-dependent manner.

### Overexpression of miR-126 augments EGCG inhibiting proliferation of osteosarcoma cells

**Figure 1:**
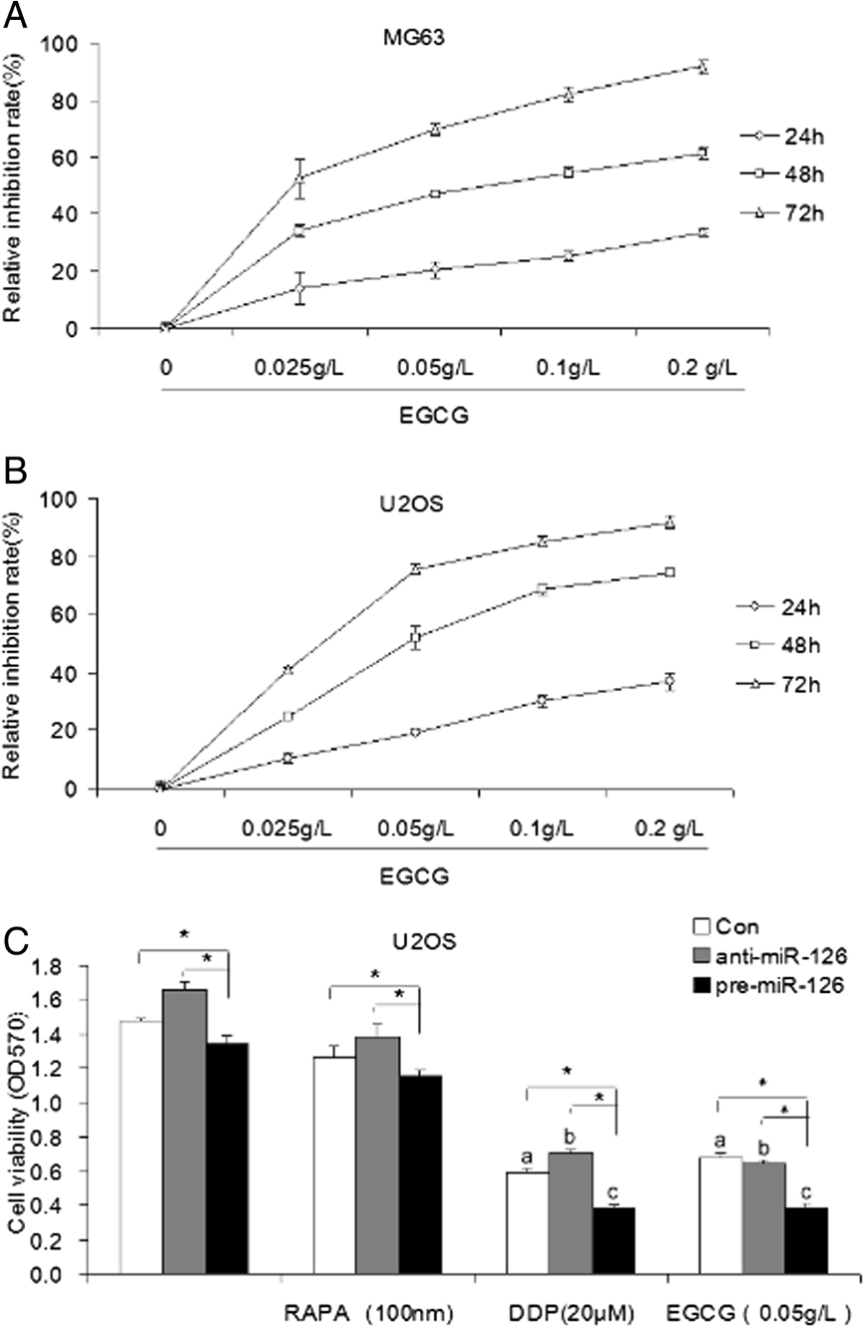
**Effect of EGCG and miR-126 on proliferation in osteosarcoma cells.** The relative inhibitory rate of EGCG on osteosarcoma MG63 **(A)** and U2OS **(B)** cells **(C)** The cell viability in U2OS cells infected with anti-miR-126, pre-miR-126, or treated with RAPA, DDP or EGCG at indicated concentration. ^*^ *P* <0.05 vs. indicated group; ^a^ *P* <0.05 vs. Con alone group; ^b^ *P* <0.05 vs. anti-miR-126 alone group; ^c^ *P* <0.05 vs. pre-miR-126 alone group. Con, control; DDP, cisplatin; EGCG, epigallocatechin-3-gallate; miR, microRNA; RAPA, rapamycin.

As showed in Figure 1C, overexpression of miR-126 decreased cell viability in U2OS cells, indicating that miR-126 serves as a suppressor in osteosarcoma cells. Moreover, RAPA, as the inhibitor of the mammalian target of rapamycin (mTOR) pathway, could not affect miR-126 inhibition of proliferation of U2OS cells, suggesting that the role of miR-126 in osteosarcoma is not dependent on the mTOR pathway.MTT assay showed that both DDP and EGCG could significantly inhibit the proliferation of osteosarcoma U2OS cells. The inhibitory effects of 0.05 g/L EGCG on U2OS cells were roughly equivalent to 20 μM DDP. Moreover, overexpression of miR-126 significantly decreased cell viability in U2OS cells treated with ECGC compared with ECGC treatment alone or combination of inhibition of miR-126 and ECGC treatment (Figure 1C).

### Overexpression of miR-126 enhances EGCG induction of apoptosis in osteosarcoma cells

**Figure 2:**
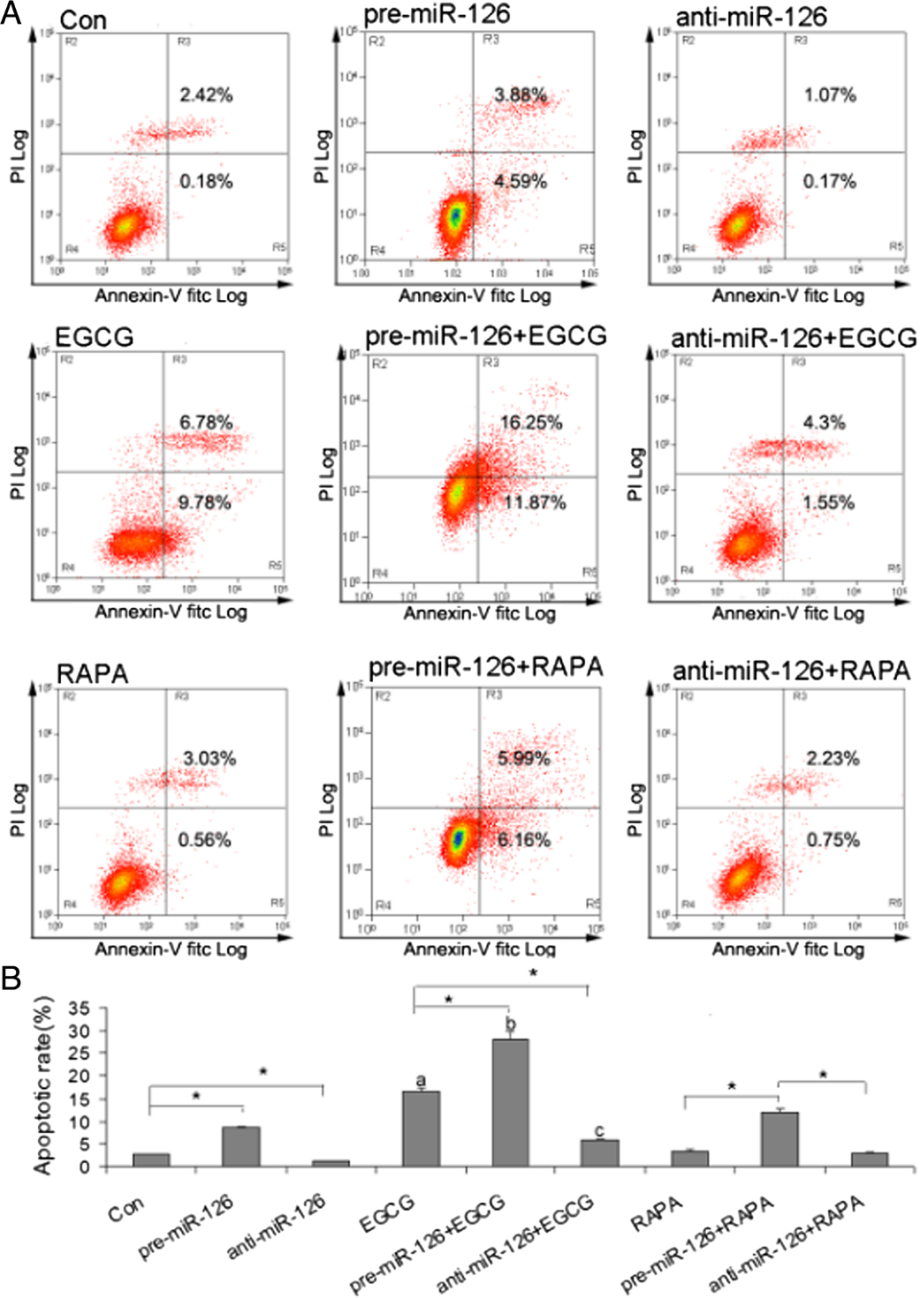
**Effect of EGCG and miR-126 on apoptosis in osteosarcoma cells. (A)** The representative images of flow cytometry analysis using Annexin V and PI staining. **(B)** The apoptotic rate in U2OS cells infected with anti-miR-126, pre-miR-126, or treated with RAPA, DDP or EGCG. ^*^ *P* <0.05 vs. indicated group; ^a^ *P* <0.05 vs. Con alone group; ^b^ *P* <0.05 vs. anti-miR-126 alone group; ^c^ *P* <0.05 vs. pre-miR-126 alone group. Con, control; DDP, cisplatin; EGCG, epigallocatechin-3-gallate; miR, microRNA; PI, propidium iodide; RAPA, rapamycin.

Flow cytometry results showed that overexpression of miR-126 could increase the apoptotic rate of osteosarcoma U2OS cells. Inhibition of miR-126 could decrease the apoptotic rate of osteosarcoma U2OS cells. The apoptosis in osteosarcoma U2OS cells induced by EGCG (0.05 g/L) was higher than that in control or overexpression of miR-126 alone group. Overexpression of miR-126 significantly enhanced EGCG-induced apoptosis in osteosarcoma U2OS cells and inhibition of miR-126 reduced EGCG-induced apoptosis in osteosarcoma U2OS cells (Figure 2).

To investigate whether the mTOR pathway is involved in miR-126 regulation of apoptosis in osteosarcoma U2OS cells, its inhibitor RAPA was used. The results showed that RAPA could not affect the apoptotic rate induced by miR-126 in U2OS cells, suggesting that the mTOR pathway is not involved in miR-126 promotion of apoptosis in osteosarcoma U2OS cells (Figure 2).

### EGCG induces G1 phase arrest in osteosarcoma cells

**Figure 3:**
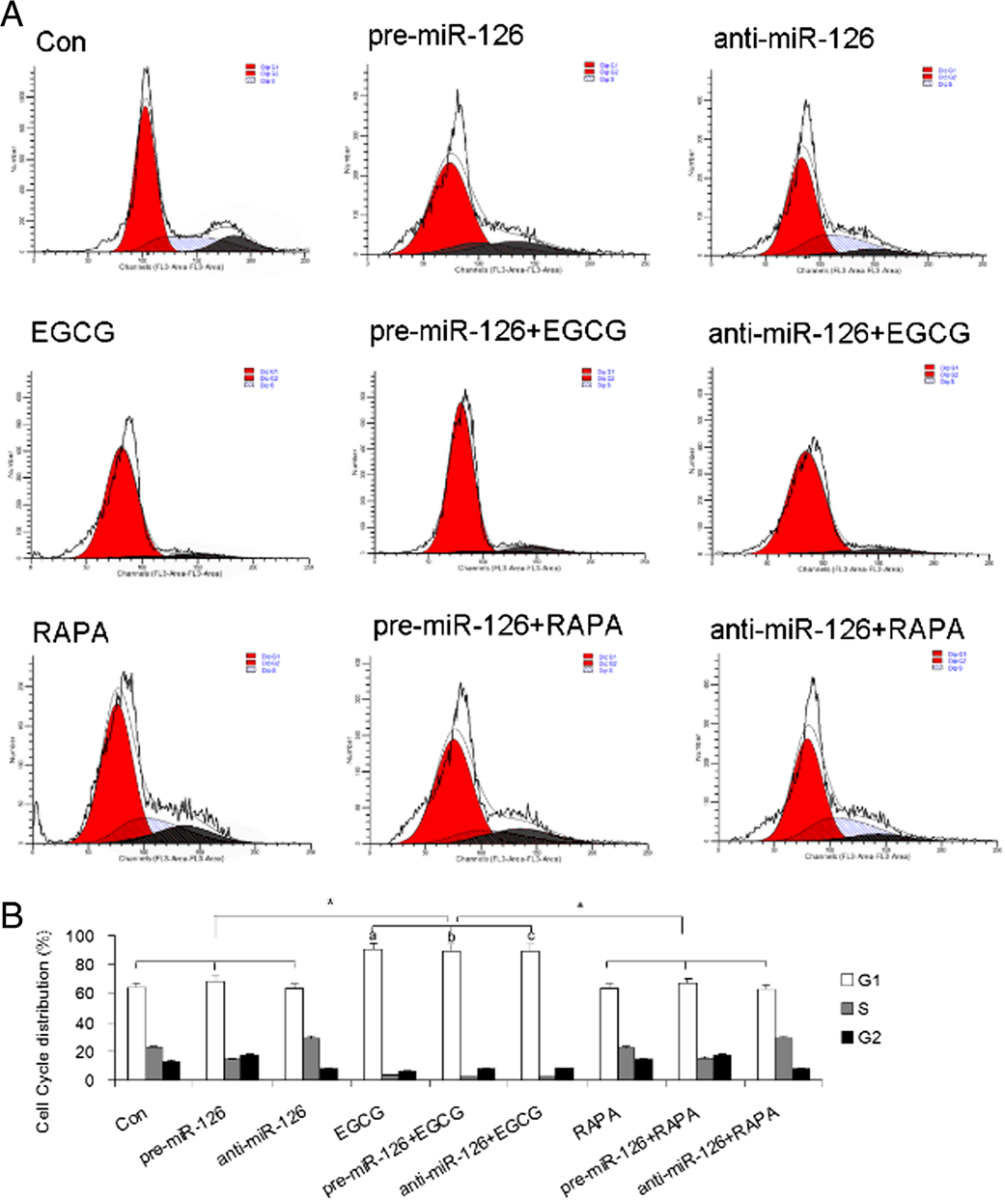
**Effect of EGCG and miR-126 on cell cycle in osteosarcoma cells. (A)** The representative images of flow cytometry analysis using PI staining. **(B)** The cell cycle distribution in U2OS cells infected with anti-miR-126, pre-miR-126, or treated with RAPA, DDP or EGCG. ^*^ *P* <0.05 vs. indicated group; ^a^ *P* <0.05 vs. Con alone group; ^b^ *P* <0.05 vs. anti-miR-126 alone group; ^c^ *P* <0.05 vs. pre-miR-126 alone group. Con, control; DDP, cisplatin; EGCG, epigallocatechin-3-gallate; miR, microRNA; PI, propidium iodide; RAPA, rapamycin.

As showed in Figure 3, overexpression of miR-126 or inhibition of miR-126 by anti-miR-126 did not have marked effects on the cell cycle G1 phase proportion in U2OS cells. And EGCG significantly increased the G1 proportion in U2OS cells and this action was not interfered by overexpression of miR-126 or inhibition of miR-126. Moreover, the ratios of G1 to S in U2OS cells were 2.8 in control, 4.8 in the pre-miR-126 group, 2.2 in the anti-miR-126 group, 26.2 in the EGCG group, 32.8 in the pre-miR-126 + EGCG group, and 32.0 in the anti-miR-126 + EGCG group. These data suggest EGCG may suppress G1/S transition in osteosarcoma cells, resulting in cell cycle G1 phase arrest, and this process is not affected by miR-126. In addition, our results also showed that combination of RAPA and miR-126 or anti-miR-126 did not affect the cell cycle G1 phase proportion in U2OS cells.

## Discussion

The natural product EGCG is the major polyphenolic constituent found in green tea. Studies suggest that EGCG is related to the potential health benefits attributed to green tea consumption [23]. The anticancer activity of EGCG has been extensively explored in past years. Although it is demonstrated that EGCG can suppress proliferation of various tumor cells, to our knowledge, articles about the roles of EGCG in osteosarcoma are few. In the present study, we confirm that EGCG can suppress proliferation of osteosarcoma MG63 and U2OS cells in a concentration-dependent and time-dependent manner; and the inhibitory effects of 0.05 g/L EGCG on U2OS cells were roughly equivalent to 20 μM DDP. These results provide novel evidence supporting development of EGCG for prevention of cancer, especially osteosarcoma.

It is well known that dysregulation of miRs will promote the malignant progression of tumor. Emerging evidences show that miRs also play an important role in the chemoresistance of cancer [24] and interference of some crucial miRs will improve the therapeutic efficacy of chemotherapeutics [25]. miR-126 is a commonly discovered loss in various cancers and its downregulation will promote proliferation, invasion, and migration in tumors [21, 22, 26]. Here, we also found miR-126 was underexpressed in MG63 and U2OS cells (data not shown), consistent with the results obtained by Yang *et al*. [19]; in the report, it was discovered that a low level of miR-126 was expressed in osteosarcoma tissues and cell lines. In addition, we found that overexpression of miR-126 resulted in inhibition of proliferation and induction of apoptosis in osteosarcoma U2OS cells. These results further confirm that miR-126 acts as a tumor suppressor in osteosarcoma.

Studies showed that miR-126 could enhance the sensitivity of non-small-cell lung cancer to adriamycin and vincristine [21] and cervical cancer cells to bleomycin [22]. In our study, we found that overexpression of miR-126 could enhance the sensitivity of osteosarcoma U2OS cells to polyphenolic EGCG. Our results showed that overexpression of miR-126 significantly enhanced the inhibitory action of EGCG on U2OS cells. Subsequently, we further verified that miR-126 could promote EGCG-induced apoptosis in U2OS cells; and EGCG-induced G1 phase arrest in U2OS cells was not apparently affected by miR-126 levels due to miR-126 itself having no marked effects on the G1 phase proportion in U2OS. The role of miR-126 identified by us in osteosarcoma cells is consistent with its performance in gastric cancer cells, that miR-126 could induce its apoptotic death but had no effects on cell cycle [27]. It suggests that the mechanisms underlying EGCG suppressing the proliferation of osteosarcoma cells mainly involve its ability to induce apoptosis and cell cycle arrest in the G1 phase, and miR-126 sensitizes U2OS cells to EGCG mainly through promotion of apoptosis.

EGCG can protect against cancer by causing cell cycle arrest and inducing apoptosis [28]. It was found that EGCG could irreversibly induce cell cycle G1 phase arrest, ultimately leading to apoptotic cell death by upregulation of WAF1/p21, KIP1/p27, INK4a/p16, and INK4c/p18, and downregulation of cyclin D1, cyclin E, cdk2, cdk4, and cdk6, irrespective of p53 status, in prostate carcinoma cells [29]. Slightly unlike this, in leukemia cells, it was demonstrated that EGCG could increase the pre-G1 phase proportion and induce apoptosis by upregulation of p53, Bax and p21 and downregulation of Bcl-2alpha [30]. These protein molecules are potential targets of EGCG in osteosarcoma cells. And miR-126 is likely a promotor of apoptotic cell death in osteosarcoma cells by regulation of PLK2, PI3KR2, Crk [27], PI3K, Akt [31], and so on.

Our results illustrate the effects of miR-126 and EGCG on proliferation, apoptosis and cell cycle distribution. However, further studies are required to elucidate the specific molecular mechanisms underlying miR-126 promotion of EGCG-induced apoptosis in osteosarcoma U2OS cells.

## Conclusions

In summary, miR-126 can enhance EGCG suppressing the proliferation of osteosarcoma cells through induction of apoptosis.

## Abbreviations

DDP: cisplatin
DMEM: Dulbecco’s modified Eagle’s medium
EGCG: epigallocatechin-3-gallate
ELISA: enzyme-linked immunosorbent assay
FITC: fluorescein isothiocyanate
IL-1: interleukin 1
miRs: microRNAs
mTOR: mammalian target of rapamycin
MTT: 3-(4,5-dimethylthiazol-2-yl)-2,5-diphenyltetrazolium bromide
PBS: phosphate-buffered saline
PI: propidium iodide
RAPA: rapamycin
SD: standard deviation.

## References

[1] Luetke A, Meyers PA, Lewis I, Juergens H (2014). Osteosarcoma treatment - where do we stand? A state of the art review. Cancer Treat Rev.

[2] Lambert JD, Sang S, Hong J, Yang CS (2010). Anticancer and anti-inflammatory effects of cysteine metabolites of the green tea polyphenol, (-)-epigallocatechin-3-gallate. J Agric Food Chem.

[3] Kalaiselvi P, Rajashree K, Bharathi Priya L, Padma VV (2013). Cytoprotective effect of epigallocatechin-3-gallate against deoxynivalenol-induced toxicity through anti-oxidative and anti-inflammatory mechanisms in HT-29 cells. Food Chem Toxicol.

[4] Cheng CW, Shieh PC, Lin YC, Chen YJ, Lin YH, Kuo DH, Liu JY, Kao JY, Kao MC, Way TD (2010). Indoleamine 2,3-dioxygenase, an immunomodulatory protein, is suppressed by (-)-epigallocatechin-3-gallate via blocking of gamma-interferon-induced JAK-PKC-delta-STAT1 signaling in human oral cancer cells. J Agric Food Chem.

[5] Pae M, Wu D (2013). Immunomodulating effects of epigallocatechin-3-gallate from green tea: mechanisms and applications. Food Funct.

[6] Braicu C, Gherman CD, Irimie A, Berindan-Neagoe I (2013). Epigallocatechin-3-Gallate (EGCG) inhibits cell proliferation and migratory behaviour of triple negative breast cancer cells. J Nanosci Nanotechnol.

[7] Ahn WS, Huh SW, Bae SM, Lee IP, Lee JM, Namkoong SE, Kim CK, Sin JI (2003). A major constituent of green tea, EGCG, inhibits the growth of a human cervical cancer cell line, CaSki cells, through apoptosis, G(1) arrest, and regulation of gene expression. DNA Cell Biol.

[8] Deng YT, Lin JK (2011). EGCG inhibits the invasion of highly invasive CL1-5 lung cancer cells through suppressing MMP-2 expression via JNK signaling and induces G2/M arrest. J Agric Food Chem.

[9] Hsieh TC, Wu JM (2009). Targeting CWR22Rv1 prostate cancer cell proliferation and gene expression by combinations of the phytochemicals EGCG, genistein and quercetin. Anticancer Res.

[10] Hwang JT, Ha J, Park IJ, Lee SK, Baik HW, Kim YM, Park OJ (2007). Apoptotic effect of EGCG in HT-29 colon cancer cells via AMPK signal pathway. Cancer Lett.

[11] Kang SU, Lee BS, Lee SH, Baek SJ, Shin YS, Kim CH (2013). Expression of NSAID-activated gene-1 by EGCG in head and neck cancer: involvement of ATM-dependent p53 expression. J Nutr Biochem.

[12] Park JS, Khoi PN, Joo YE, Lee YH, Lang SA, Stoeltzing O, Jung YD (2013). EGCG inhibits recepteur d’origine nantais expression by suppressing Egr-1 in gastric cancer cells. Int J Oncol.

[13] Trudel D, Labbe DP, Araya-Farias M, Doyen A, Bazinet L, Duchesne T, Plante M, Gregoire J, Renaud MC, Bachvarov D, Têtu B, Bairati I (2013). A two-stage, single-arm, phase II study of EGCG-enriched green tea drink as a maintenance therapy in women with advanced stage ovarian cancer. Gynecol Oncol.

[14] Chen D, Pamu S, Cui Q, Chan TH, Dou QP (2012). Novel epigallocatechin gallate (EGCG) analogs activate AMP-activated protein kinase pathway and target cancer stem cells. Bioorg Med Chem.

[15] Honicke AS, Ender SA, Radons J (2012). Combined administration of EGCG and IL-1 receptor antagonist efficiently downregulates IL-1-induced tumorigenic factors in U-2 OS human osteosarcoma cells. Int J Oncol.

[16] Ji F, Zhang H, Wang Y, Li M, Xu W, Kang Y, Wang Z, Wang Z, Cheng P, Tong D, Li C, Tang H (2013). MicroRNA-133a, downregulated in osteosarcoma, suppresses proliferation and promotes apoptosis by targeting Bcl-xL and Mcl-1. Bone.

[17] Fan L, Wu Q, Xing X, Wei Y, Shao Z (2012). MicroRNA-145 targets vascular endothelial growth factor and inhibits invasion and metastasis of osteosarcoma cells. Acta Biochim Biophys Sin.

[18] Han K, Zhao T, Chen X, Bian N, Yang T, Ma Q, Cai C, Fan Q, Zhou Y, Ma B (2014). microRNA-194 suppresses osteosarcoma cell proliferation and metastasis in vitro and in vivo by targeting CDH2 and IGF1R. Int J Oncol.

[19] Yang C, Hou C, Zhang H, Wang D, Ma Y, Zhang Y, Xu X, Bi Z, Geng S (2014). miR-126 functions as a tumor suppressor in osteosarcoma by targeting Sox2. Int J Mol Sci.

[20] Zhao G, Cai C, Yang T, Qiu X, Liao B, Li W, Ji Z, Zhao J, Zhao H, Guo M, Ma Q, Xiao C, Fan Q, Ma B (2013). MicroRNA-221 induces cell survival and cisplatin resistance through PI3K/Akt pathway in human osteosarcoma. PLoS One.

[21] Zhu X, Li H, Long L, Hui L, Chen H, Wang X, Shen H, Xu W (2012). miR-126 enhances the sensitivity of non-small cell lung cancer cells to anticancer agents by targeting vascular endothelial growth factor A. Acta Biochim Biophys Sin.

[22] Yu Q, Liu SL, Wang H, Shi G, Yang P, Chen XL (2013). miR-126 Suppresses the proliferation of cervical cancer cells and alters cell sensitivity to the chemotherapeutic drug bleomycin. Asian Pac J Cancer Prev.

[23] Singh BN, Shankar S, Srivastava RK (2011). Green tea catechin, epigallocatechin-3-gallate (EGCG): mechanisms, perspectives and clinical applications. Biochem Pharmacol.

[24] Xu X, Wells A, Padilla MT, Kato K, Kim KC, Lin Y (2014). A signaling pathway consisting of miR-551b, catalase and MUC1 contributes to acquired apoptosis resistance and chemoresistance. Carcinogenesis.

[25] Weng H, Huang H, Dong B, Zhao P, Zhou H, Qu L (2014). Inhibition of miR-17 and miR-20a by oridonin triggers apoptosis and reverses chemoresistance by derepressing BIM-S. Cancer Res.

[26] Frampton AE, Krell J, Jacob J, Stebbing J, Castellano L, Jiao LR (2012). Loss of miR-126 is crucial to pancreatic cancer progression. Expert Rev Anticancer Ther.

[27] Liu LY, Wang W, Zhao LY, Guo B, Yang J, Zhao XG, Hou N, Ni L, Wang AY, Song TS, Huang C, Xu JR (2014). Mir-126 inhibits growth of SGC-7901 cells by synergistically targeting the oncogenes PI3KR2 and Crk, and the tumor suppressor PLK2. Int J Oncol.

[28] Ahmad N, Feyes DK, Nieminen AL, Agarwal R, Mukhtar H (1997). Green tea constituent epigallocatechin-3-gallate and induction of apoptosis and cell cycle arrest in human carcinoma cells. J Natl Cancer Inst.

[29] Gupta S, Hussain T, Mukhtar H (2003). Molecular pathway for (-)-epigallocatechin-3-gallate-induced cell cycle arrest and apoptosis of human prostate carcinoma cells. Arch Biochem Biophys.

[30] Harakeh S, Abu-El-Ardat K, Diab-Assaf M, Niedzwiecki A, El-Sabban M, Rath M (2008). Epigallocatechin-3-gallate induces apoptosis and cell cycle arrest in HTLV-1-positive and -negative leukemia cells. Med Oncol.

[31] Sui XQ, Xu ZM, Xie MB, Pei DA (2014). Resveratrol inhibits hydrogen peroxide-induced apoptosis in endothelial cells via the activation of PI3K/Akt by miR-126. J Atheroscler Thromb.

